# Effects of smoking cessation on plasma clozapine concentrations in male patients with schizophrenia during the COVID-19 pandemic

**DOI:** 10.3389/fpsyt.2023.1256264

**Published:** 2023-09-15

**Authors:** Botao Ma, Hongzhen Fan, Siyuan Qi, Fude Yang, Huimei An

**Affiliations:** Psychiatry Research Center, HuiLongGuan Clinical Medical School, Beijing HuiLongGuan Hospital, Peking University, Beijing, China

**Keywords:** clozapine, schizophrenia, smoking cessation, therapeutic drug monitoring, plasma concentrations

## Abstract

**Introduction:**

This study aimed to investigate the effect of smoking cessation on plasma clozapine (CLO) concentrations in long-term hospitalized Chinese male patients with schizophrenia treated with CLO during the COVID-19 pandemic.

**Methods:**

Therapeutic drug monitoring (TDM) data for CLO were collected at Beijing HuiLongGuan Hospital between December 1, 2019 (before smoking cessation) and January 31, 2020 (after smoking cessation) in this retrospective study. Fifty-three male smokers and inpatients with schizophrenia who were treated with CLO were included. Plasma concentrations of CLO were measured using high-performance liquid chromatography–tandem mass spectrometry. The fagerstrom test for nicotine dependence (FTND) was used to assess smoking behavior.

**Results:**

The plasma CLO concentrations and dose-corrected plasma CLO concentrations were significantly increased by 29.3 and 23.5%, respectively, after smoking cessation.

**Discussion:**

The results suggested that clinicians and pharmacists should adjust the CLO dose based on changes in smoking status in patients stabilized with CLO during the COVID-19 pandemic. Careful TDM for CLO should be performed prior to dose adjustment，to reduce the increased risk of smoking cessation induced side effects, especially for older patients receiving multiple medications.

## 1. Introduction

Clozapine (CLO), a unique antipsychotic drug, is widely used to treat schizophrenia, especially refractory schizophrenia (1). The initial dose of 12.5 mg is gradually increased to 300–400 mg/day, with a maximum licensed dose of 900 mg/day (2). The adverse effects of CLO may be dose-dependent and include sedation, drowsiness, dizziness, headache, excessive salivation, postural hypotension, hyperthermia, constipation, myocarditis, weight gain, cardiomyopathy, and seizures (3–5).

The therapeutic drug monitoring (TDM) of CLO can help oversee adherence, improve efficacy, and reduce adverse effects. According to the consensus guidelines of Arbeitsgemeinschaft für Neuropsychopharmakologie und Pharmakopsychiatrie, CLO is strongly recommended for level 1 TDM. The recommended therapeutic reference range for CLO is 350–600 ng/mL, while the laboratory alert level is 1,000 ng/mL (6).

CLO is metabolized via *N*-demethylation, aromatic hydroxylation, *N*-oxidation, and other pathways (7). It is primarily metabolized by CYP1A2 to its major metabolite *N*-desmethylclozapine (8). Other cytochrome P450 (CYP) enzymes including CYP2D6, CYP3A4, CYP2C9, and CYP2C19, flavin monooxygenase 3, UDP glucuronosyltransferase 1A4, and the *P*-glycoprotein transporter ABCB1 are also involved in CLO metabolism (2, 8–10).

Previous studies reported that smoking significantly affects CLO metabolism. Plasma CLO concentrations decrease by approximately 20–40% in smokers versus non-smokers (11–13). A possible explanation for the lower plasma concentrations in smokers is that polycyclic aromatic hydrocarbons (PAHs) released during tobacco smoking induce CYP enzymes, particularly CYP1A2, resulting in more rapid CLO clearance (14). Therefore, smokers require higher doses of CLO (15). Smoking cessation can reverse smoking-induced CYP1A2 enzyme activity to normal levels, markedly increasing plasma CLO concentrations (16). Due to the narrow therapeutic ratio of CLO, increasing plasma drug concentrations after smoking cessation may cause serious side effects. Therefore, TDM of CLO is required for patients after smoking cessation.

Owing to the coronavirus disease 2019 (COVID-19) outbreak in Beijing, wards were forced to implement closed management practices on December 20, 2019. None of the inpatients were allowed to smoke outside the ward to prevent cross-infection; thus, a smoking cessation study sample was generated. Previous studies demonstrated significant ethnic differences in CLO pharmacokinetics. For example, Asian populations require only half the CLO dose used by Americans to obtain the same blood concentrations. However, most previous studies of the effect of smoking cessation on CLO metabolism were case reports, and the subjects were from European and American populations but rarely from Chinese populations. As the proportion of male smokers is significantly higher than female smokers in the Chinese population, we included only male inpatients (17). Therefore, this study aimed to investigate the effects of smoking cessation on plasma CLO concentrations in long-term hospitalized Chinese male patients with schizophrenia treated with CLO during the COVID-19 pandemic in China.

## 2. Methods

### 2.1. Participants

This was a retrospective cross-sectional study. TDM data for CLO were collected at Beijing HuiLongGuan Hospital between December 1, 2019 (before smoking cessation) and January 31, 2020 (after smoking cessation). Fifty-three male smokers and inpatients with schizophrenia treated with CLO were included. All participants were Han Chinese aged 18–85 years. The patients were stable and received a fixed dose of CLO (50–550 mg/day) orally for at least 3 months. All participants had a smoking history of more than 1 year and were current smokers of more than five cigarettes per day. Demographic and clinical information, including sex, age, weight, psychiatric diagnosis, co-medication, daily dose, date of dose adjustment, time of drug intake, and date of blood sampling, were obtained from the patients’ medical records. Patients who received drugs with significant effects on CLO metabolism, those who received sustained-release CLO, and those with known physical disorders or substance dependence were excluded. This study was approved by the Review Board of Beijing HuiLongGuan Hospital. Written informed consent was not required for the TDM analysis since it is a routine clinical blood test.

### 2.2. Blood sampling

Blood samples for TDM of CLO were collected under fasting conditions in the morning, usually between 7 a.m. and 8 a.m., approximately 12 h after the last CLO dose. They were immediately centrifuged at 3000× *g* at 4°C for 10 min. The plasma samples were collected and stored at −20°C for the determination of CLO concentrations, which was completed within 1 week.

### 2.3. Determination of plasma CLO concentrations

Steady-state plasma CLO concentrations were measured using high-performance liquid chromatography–tandem mass spectrometry (HPLC-MS/MS). One hundred microliters of plasma was added to 300 μL of acetonitrile solution containing d4-CLO (50 ng/mL), vortexed and mixed for 30 s, and centrifuged at 14,000 rpm for 10 min. One hundred fifty microliters of the supernatant was subjected to HPLC-MS/MS analysis. HPLC was performed using an LC20A system (Shimadzu, Kyoto, Japan). Sample separation was performed on a phenyl–hexyl column (Phenomenex; 50 × 4.6 mm, 2.6 μm) with a gradient elution program in 4.5 min. Ammonium formate aqueous solution (5 mM) with 0.05% formic acid (A) and acetonitrile with 0.05% formic acid (B) were used as the mobile phase with a flow rate of 0.6 mL/min at 40°C. The gradient elution was run as follows: 15 to 95% mobile phase B from 0 to 2.4 min, 95% mobile phase B from 2.4 to 3.4 min, and 15% mobile phase B from 3.4 to 4.5 min. MS/MS was performed using an AB Sciex Triple Quad 4,500 system (Applied Biosystems, Foster City, CA, USA). The electrospray ionization positive ion mode with multiple reaction monitoring was used for CLO detection using the following parameters: spray voltage, 5,000 V; ion source temperature, 550°C; collision gas, 8 psi; curtain gas, 40 psi; ion source gas 1, 55 psi; and ion source gas 2, 50 psi. The quantitative ion pairs were 327.3/270.0 for CLO and 331.4/272.1 for CLO-d4. The collision energies were 48 and 33 V for CLO and CLO-d4, respectively. The declustering, entrance, and collision exit potentials of CLO and CLO-d4 were 80, 10, and 10 V, respectively. The standard curves of CLO were linear in the range of 10–1,000 ng/mL. All precision and accuracy values were less than ±5%. The detection limit for CLO was 0.25 ng/mL. The lower limit of quantification for CLO was 10 ng/mL. The average recovery rate of CLO was 98%.

### 2.4. Smoking behavior assessment

The Fagerstrom Test for Nicotine Dependence was used to assess the participants’ smoking behavior, including smoking history and average number of cigarettes currently smoked per day. This information was objectively verified using the medical records and statements from other family members. In the present study, smoker status was defined as a history of smoking for more than 1 year and smoking more than five cigarettes per day.

### 2.5. Statistical analyses

The data analyses were performed using SPSS 20.0. Descriptive statistics are expressed as the mean ± standard deviation for continuous variables and percentages for categorical variables. The Kolmogorov–Smirnov test was used to examine normality. Paired t-tests were used to compare differences in dose, plasma CLO concentration, and dose-corrected plasma CLO concentration before and after smoking cessation. Statistical significance was set at *p* < 0.05.

## 3. Results

### 3.1. Demographic and clinical characteristics

Demographic and clinical characteristics of the participants are shown in Table 1. All 53 male schizophrenia inpatients were from the Han population, with a mean age of 57.5 ± 9.3 years, duration of illness of 35.8 ± 10.6 years, and mean daily smoking of 14.3 ± 11.9. The daily CLO dose positively correlated with the plasma concentrations of CLO (*r* = 0.423, *p* = 0.002). Before smoking cessation, the median of daily CLO dose was 225 (50–425) mg, the median of plasma CLO concentrations were 392 (167–797) ng/ml, with 5 (9.4%) patients having plasma CLO concentrations above the upper limit of the therapeutic reference range, i.e., 600 ng/mL. No patient had plasma CLO concentrations above the laboratory alert concentration of 1,000 ng/mL. After smoking cessation, the median of daily CLO dose was 200 (75–425) mg, the median of plasma CLO concentrations were 482 (210–1,174) ng/ml, with 14 (26.4%) patients having plasma CLO concentrations ≥600 ng/mL, and 2 (3.7%) patients having plasma CLO concentrations ≥1,000 ng/mL. Approximately 38.3% of the patients used only CLO, 40% used a combination of antipsychotics, 31.2% used a combination of benzodiazepines, 8.3% used a combination of antidepressants, and about 6.7% used a combination of mood stabilizers.

**Table 1:** Demographic and clinical characteristics of participants.

Parameters	*n* = 53
Age (years)	57.5 ± 9.3
Education(years)	11.3 ± 2.6
BMI(kg/m^2^)	24.4 ± 3.8
Han Chinese	100%
Age of onset	22.4 ± 5.3
Duration of illness	35.8 ± 10.6
Daily smoking (cigarettes)	14.3 ± 11.9
Co-medication	
None	38.3%
Antipsychotics	40.0%
Antidepressants	8.3%
Benzodiazepines	31.2%
Emotional stabilizer	6.7%

### 3.2. Comparison of plasma CLO concentrations before and after smoking cessation

**Figure 1:**
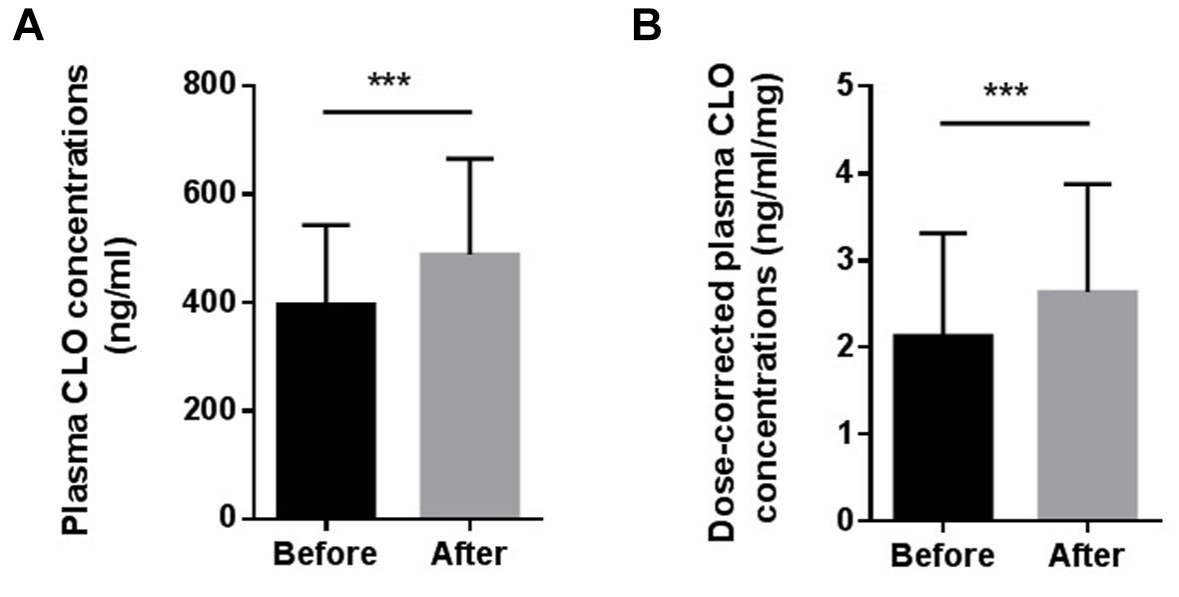
Comparison of the plasma CLO concentrations **(A)** and dose-corrected plasma CLO concentrations **(B)** before and after smoking cessation, ***indicates *p* < 0.001.

Figure 1 presents the differences in the plasma CLO concentrations and dose-corrected plasma CLO concentrations before and after smoking cessation. The daily dose of CLO decreased slightly after the smoking cessation, but there was no significant difference before or after smoking cessation (219.6 ± 93.1 mg/day for before smoking cessation *Vs* 214.7 ± 84.0 mg/day for after smoking cessation, *t* = 1.940, *p* = 0.058). The plasma CLO concentrations after smoking cessation was significantly higher than before smoking cessation (396.9 ± 146.5 ng/mL for before smoking cessation *Vs* 512.4 ± 211.4 ng/mL for after smoking cessation, *t* = −6.491, *p* < 0.001). This was significantly increased by 29.3%. Similarly, The dose corrected plasma CLO concentrations after smoking cessation were also significantly higher than before smoking cessation (2.13 ± 1.18 ng/mL/mg for before smoking cessation *Vs* 2.63 ± 1.24 ng/mL/mg for after smoking cessation, *t* = −5.164, *p* < 0.001). It was significantly increased by 23.5%.

## 4. Discussion

To the best of our knowledge, this study firstly investigated the effect of smoking cessation on plasma CLO concentrations in long-term hospitalized Chinese male patients with schizophrenia treated with CLO during the COVID-19 pandemic in China. In the present study, plasma CLO concentrations and dose-corrected plasma CLO concentrations were significantly increased by 29.3 and 23.5%, respectively, after smoking cessation.

Smoking status is an important factor influencing CLO metabolism. The present study found that plasma CLO concentrations and dose-corrected plasma CLO concentrations of hospitalized patients increased significantly by 29.3 and 23.5%, respectively, approximately 2 weeks after the smoking ban. Theoretically, this result could be well explained by Faber and Fuhr’s study, which determined the time response of CYP1A2 activity following heavy smoking cessation and found that CYP1A2 activity decreased by 20 and 36% on days 2 and 7 after smoking cessation and reached a new steady state after 1 week (18). Smoking cessation can reverse PAH-induced CYP1A2 activity to normal levels and markedly increase plasma CLO concentrations in patients administered the same dose of CLO as smokers (16).

Several observational clinical studies conducted in different countries reported similar results. For example, a recent study by Japanese scholars Tsukahara et al., who investigated plasma CLO concentrations before and after smoking cessation in 14 Japanese inpatients with schizophrenia who were being treated with CLO when a non-smoking policy was implemented in a psychiatric hospital, found that the mean plasma CLO concentrations and dose-corrected plasma CLO concentrations were significantly increased by 53.2 and 42.5%, respectively. Of them, four experienced serious side effects such as amnesia, drooling, and myoclonus owing to the increased plasma CLO concentrations (19). Similarly, a study from the United States analyzed the CLO concentrations changes in 11 patients treated with stabilized CLO doses at Oregon State Hospital before and after the implementation of a smoking ban and found 57.4% increase in CLO concentrations and 82.1% increase in dose-corrected plasma CLO concentrations upon smoking cessation (12).

In addition, several case reports have described increases in side effects accompanied by increases in plasma CLO concentrations following smoking cessation in patients receiving CLO. For example, Zullino et al. reported a case of a 37-year-old man with an 8-year history of paranoid schizophrenia treated with CLO at 700 mg/day developed confusion 1 month after smoking cessation. His plasma CLO concentrations were 350 ng/mL before smoking cessation and 1,328 ng/mL after smoking cessation. His dose-corrected plasma CLO concentrations also increased from 0.5 ng/mL/mg before smoking cessation to 1.89 ng/mL/mg after smoking cessation. When the CLO dose was reduced to 500 mg/day, the confusion disappeared within 1 week and the plasma CLO concentration returned to the pre-smoking cessation level (20). Similarly, Derenne and Baldessarini reported the case of a 28-year-old white woman with severe psychiatric illness treated with CLO 450 mg/day who developed sedation, confusion, muscle spasms, dry mouth, dizziness, sluggish pupils, and mild delirium upon abruptly stopping her habit of heavy smoking. Her serum CLO concentrations were 1,615 ng/mL while not-smoking and 338 ng/mL at 350 mg/day while smoking. Mean dose-corrected plasma CLO concentrations were 4.65 ng/mL/mg while not smoking compared to 2.25 ng/mL/mg while smoking. The symptoms were rapidly relieved when the CLO dose was reduced (21). Bondolfi et al. reported a 51-year-old white male with paranoid schizophrenia who was treated with CLO and developed severe sedation and fatigue 2 weeks after smoking cessation. His dose-corrected plasma CLO concentrations increased from 0.57 ng/mL/mg before smoking cessation to 1.90 ng/mL/mg after smoking cessation. These adverse effects diminished as the CLO dose decreased (22).

In the present study, it was also found that before smoking cessation, only 9.4% patients had plasma CLO concentrations above the upper limit of the therapeutic reference range, i.e., 600 ng/mL, and no patient had plasma CLO concentrations higher than the laboratory alert concentration of 1,000 ng/mL. However, after smoking cessation, the proportion of patients with plasma CLO concentrations ≥600 ng/mL increased to 26.4%, while 3.7% of patients had plasma CLO concentrations ≥1,000 ng/mL. These results were similar to the results reported by cormac et al., they retrospectively collected data on CLO doses and plasma concentrations from 3 months before and 6 months after the smoking ban at Lambton Hospital in the UK and evaluated the effect of smoking cessation on psychiatric patients taking CLO. They found that only 4.2% of smoking patients had plasma CLO concentrations ≥1,000 mg/L before the smoking ban, a percentage that increased to 41.7% within the 6-month period after the smoking ban despite dose reduction (23). All these results further suggested that smoking cessation may significantly increase blood CLO concentrations and lead to an increased risk of side effects.

However, the mean 29.3% increase in plasma CLO concentration and 23.5% increase in dose-corrected plasma CLO concentrations in the Chinese male inpatients with schizophrenia observed in this study was lower than those reported in previous studies from Japan, Europe and the United States. Previous studies suggested significant ethnic differences in the metabolism of CLO between Caucasians and Asians and that the induction effect of PAHs on CYP1A2 enzyme activity in Asians is weaker than that in Caucasians (24, 25). Furthermore, the number of cigarettes smoked per day may affect the extent of CYP1A2 induction, and smoking 20 cigarettes per day appears to produce the maximum induction (26). In addition to ethnicity and number of cigarettes smoked per day, sex, age, and co-medication may explain this difference. This study enrolled only male inpatients a mean age of 57.5 ± 9.3 years, a sample that was older than those in other studies. In addition, approximately 60% of the patients were receiving one or more other medications that may affect CYP1A2 enzyme activity (25). All of the reasons mentioned above can explain the numerical differences in the results among studies.

This study has several limitations. First, it did not investigate the incidence of adverse effects after smoking cessation owing to its retrospective design. Second, it is a pity that in the present study we could not obtain plasma *N*-desmethylclozapine (NDMC) concentrations, although the plasma concentrations ratio of NDMC to CLO is important index for the assessment of CLO metabolism by CYPs. These aspects will be investigated in future prospective studies. Third, as age affects CLO metabolism and the mean patient age in the present study was close to 60 years, the results should be interpreted with caution, especially when examining younger patients.

In conclusion, the study found that plasma CLO concentrations were significantly increased by 29.3% after smoking cessation in long-term hospitalized Chinese male patients with schizophrenia treated with CLO during the COVID-19 pandemic in China. Therefore, to reduce the increased risk of smoking cessation-induced side effects, clinicians and pharmacists should adjust the CLO dose based on changes in smoking status. In addition, careful TDM of CLO should be performed prior to dose adjustment, especially in older patients receiving multiple medications.

## Data availability statement

The data analyzed in this study is subject to the following licenses/restrictions: The data are not publicly available due to personal privacy but are available from the corresponding author on reasonable request. Requests to access these datasets should be directed to anhuimei_teacher@163.com.

## Ethics statement

The studies involving humans were approved by the Review Board of Beijing HuiLongGuan Hospital. The studies were conducted in accordance with the local legislation and institutional requirements. The ethics committee/institutional review board waived the requirement of written informed consent for participation from the participants or the participants’ legal guardians/next of kin because Written informed consent was not required for the TDM analysis for clozapine since it is a routine clinical blood test.

## Author contributions

BM: Data curation, Investigation, Resources, Writing – original draft, Writing – review & editing. HF: Data curation, Formal analysis, Methodology, Validation, Writing – review & editing. SQ: Data curation, Methodology, Writing – review & editing. FY: Project administration, Resources, Supervision, Writing – review & editing. HA: Conceptualization, Data curation, Project administration, Supervision, Writing – original draft, Writing – review & editing.

## Funding

The author(s) declare financial support was received for the research, authorship, and/or publication of this article. This study was supported by grants from the Beijing Municipal Natural Science Foundation (7182074 and 7214238) and Beijing Municipal Science & Technology Commission no. Z191100006619020. The supporters had no role in the design, analysis, interpretation, or publication of this study.

## Conflict of interest

The authors declare that the research was conducted in the absence of any commercial or financial relationships that could be construed as a potential conflict of interest.

## Publisher’s note

All claims expressed in this article are solely those of the authors and do not necessarily represent those of their affiliated organizations, or those of the publisher, the editors and the reviewers. Any product that may be evaluated in this article, or claim that may be made by its manufacturer, is not guaranteed or endorsed by the publisher.
